# Language Communication Skills in Health and Social Care Workers

**Published:** 2019-04

**Authors:** Monika MACKINOVA, Pavol KOPINEC, Jan HOLONIC, Jaroslav STANCIAK

**Affiliations:** Department of Social Work, Faculty of Education, Comenius University, Bratislava, Slovak Republic

## Dear Editor-in-Chief

Social workers and healthcare professionals who work e.g. with refugees and migrants from distinctly non-European ethnic backgrounds are very much aware of the push-pull factors and conflictive family relationships that could arise as children learn the language of their new country, habits, and styles which are likely to be different from the ones used at home. The communication issues are the aim several studies (1, 2). In Slovakia have always been offered foreign languages within the primary and secondary school curriculum and in the necessary general education of universities.

Language communication skills of health and social workers impact on the quality of health care. Quality of health care is multifaceted concept (3, 4).

The main goal of research was to analyze the linguistic competence of students in undergraduate studies, their practical skills, and experience with the application of a foreign language in practice. Another goal was to estimate language skills in employees in healthcare and social services, in order to identify the linguistic competence in academic and professional spheres of health and social sector.

Survey targeted undergraduate students and people already working in either healthcare or social work. The survey was carried out from November 2017 to February 2018. The sample consisted of 200 respondents from the field of social work and health in undergraduate studies and 200 professionals from social work and healthcare.

Of the 100 respondents surveyed in healthcare undergraduate studies, only 10 respondents did not speak any foreign language, 90 respondents can speak one language 19 respondents speak two or more languages. In contrast, 25 students in undergraduate studies of social work do not speak any foreign language and 75 respondents have the average level of communication in a foreign language, of which 15 respondents speak two or more languages.

All respondents have been informed about the research related information (details of the nature and purpose of the research, its duration etc.), their rights and benefits of participation to voluntarily decide and make informed decision whether or not to participate as a research subject. Information by the participants have been anonymous with assured confidentiality.

From 45 respondents under 35 yr old already working in healthcare, 12 respondents do not have any foreign language skills and 33 respondents can speak one foreign language and 15 respondents are able to speak two or more foreign languages. Same category respondents from social work 22 respondents were surveyed, five respondents do not have any other language knowledge, 17 respondents speak one foreign language and 11 can speak two or more foreign languages. In the age group over 35 yr old in healthcare, from 55 respondents 17 cannot speak any other language, 38 respondents know at least one world language and 13 respondents is able to communicate in two or more foreign languages. In social work from 78 respondents over 35 yr old surveyed, 38 have no appropriate language skills, 40 respondents speaks one foreign language, and 18 two or more foreign languages. Health care workers have better language competence in comparison to workers in the social field.

Question about using a foreign language in practice: 40 students in the medical field answered that they sometimes apply their language skills in the study, 60 respondents never apply their language skills. A surprising finding is that 88 students of social work never use foreign language in their education 12 of them only sometimes.

From 45 respondents under 35 yr old working in healthcare 10 respondents use a foreign language in the profession, 22 respondents sometimes and 10 respondents do not use foreign language at work. In social work in the same category, from 22 respondents surveyed 10 respondents apply foreign language skills sometimes in their profession and 11 respondents do not need or do not use a foreign language at work. Based on these answers, people in healthcare have better language skills and use foreign languages more often in comparison to those in social work.

When asked whether employer allows increasing linguistic competence, we found surprising results. Only students in undergraduate studies as well as in health and social areas have the opportunity to improve their level of knowledge of a foreign language, probably also by attending courses at university. In contrast, respondents in work in both sectors do not have the option of increasing their language level, except in their free time.

Students in both areas are very open to improve their language skills. However, older respondents in the social work do not seem to have an effort to improve their language for better performance at work, 19 from 59 respondents answered, that they do not want to improve their skills.

Professionals not always have the opportunity to do so after their university studies and sometimes also social services providers do not have knowledge in recognizing of importance of language and cultural competences of their employees.

## References

[1] Radović V Ćurčić L (2012). The Opportunities of Crises and Emergency Risk Communication in Activities of Serbian Public Health Workforce in Emergencies. Iran J Public Health, 41 (10): 15–23. PMC3494226 23308348

[2] Azmawati MN Aniza I Ali M (2013). Evaluation of Communication for Behavioral Impact (COMBI) Program in Dengue Prevention: A Qualitative and Quantitative Study in Selangor, Malaysia. Iran J Public Health, 42 (5): 538–539. 23802114 PMC3684465

[3] Samohyl M Nadazdyova A Hirosova K (2017). Patients Satisfaction with Dental Care in the Slovak Republic: A Cross-sectional Questionnaire Study. Iran J Public Health, 46 (8): 1143–1144. 28894719 PMC5575397

[4] Samohyl M (2014). Quality of Health Care. Post-grad Med, 16 (8): 887–892.

